# Internal Parasites of Pigs and Worm Control Practices in Bamboutos, Western Highlands of Cameroon

**DOI:** 10.1155/2018/8242486

**Published:** 2018-11-21

**Authors:** Marc K. Kouam, Fabrice D. Ngueguim, Vaia Kantzoura

**Affiliations:** ^1^Department of Animal Production, Faculty of Agronomy and Agricultural Sciences, P.O. Box 188, Dschang, Cameroon; ^2^Center for Research on Filariasis and Other Tropical Diseases (CRFilMT), P.O. Box 5797, Yaoundé, Cameroon; ^3^Veterinary Research Institute, HAO-Demeter, NAGREF Campus, Thessaloniki, Greece

## Abstract

Internal parasites are limiting factors to successful, sustainable livestock production. Knowledge on how they are dealt with is important to prevent resistance to anthelmintics. The aim of this study was to describe the internal parasitism of indoor pigs in Bamboutos Division in Cameroon, as well as the attendant worm control practices. Thus, 324 pigs from 50 small scale farms were sampled for feces which were qualitatively and quantitatively examined for parasite eggs, cysts, or oocysts. Data on worm control practices were also collected. The overall prevalence was 74.7% (95 % Confidence Interval (CI): 69.6–79.3%) and the overall mean egg/oocyst per gram of feces (epg/opg) was 304.1±1218.0. The following parasites were found: Strongylid parasites (58.6%; epg= 105.0±134.7); Coccidia (26.9; opg=517.2± 1862.1);* Strongyloides ransomi* (25.9%; epg=61.9± 40.8);* A. suum* (3.7%. epg=50±0);* Metastrongylus* sp (0.9%; epg=50±0);* Trichuris suis* (0.9%; epg=50±0); and* Macracanthorhynchus hirudinaceus* (0.62%; epg=50±0). Single to septuple infestations occurred. The majority of farmers resorted to modern veterinary services (64%) and mostly used conventional drugs (88%). Internal parasitism was associated with the person in charge of animal health, the implementation of a prophylaxis program on the farm, and the annual deworming frequency. The implementation of a prophylaxis program significantly reduced the overall egg/oocyst load while high treatment frequency (more than thrice a year) did not, indicating that prophylaxis measures such as general hygiene must be reinforced in pig herds in the country, and the treatment frequency reduced as much as possible to prevent the selection of anthelmintic resistance.

## 1. Introduction

Internal parasites of pigs in Cameroon are poorly documented. To our knowledge, the most recent data date back to 17 years ago in outdoor pigs in Menoua in the West region of the country [1]. Considering the growth in pig population for instance from 1.7000,000 heads in 2009 [2] to 2, 896, 271 heads in 2012 [3], shortage of basic data on pig parasitism is rather surprising. The warm temperature and humidity in the tropics [4] as well as the poor management practices on pig farms in sub-Saharan Africa [5] invariably cause pigs to be infested and to carry heavy burdens of gastrointestinal parasites. Whatever the production system that may be adopted in these sub-Saharan countries, parasitism is likely to occur and to constitute a hindrance to efficient and profitable pig production [6]. Indeed, extensively managed pigs are reported to harbor intestinal helminthes and protozoans [7] while pigs raised in intensive operations though thought to be less prone to gastrointestinal infestation are infested as well; the large roundworm (*Ascaris suum*), whipworm (*Trichuris suis*), the nodular worms (*Oesophagostomum* sp), and protozoa (Coccidia) are often found in intensive pig production [8, 9].

The harm caused by pig parasites is well known. Parasites, especially gastrointestinal parasites, prevent a productive pig husbandry by robbing the essential nutrients required for optimum reproduction and growth, or by causing lesions leading to condemnation during inspection of organs [10], diarrhea [4, 11], poor feed conversion [12], and even death [12].

In the same line, with the phenomenon of drug resistance reported to occur worldwide [13–15], it is important to have a clear picture of how diseases, particularly internal parasitism, are handled in pig husbandry. Knowledge on parasite control practices is also necessary to design strategies to mitigate the harmful effect of parasitism in livestock. Within the country, Bamboutos is known as one of the divisions where intensive and extensive pig farming are practiced [16], since intensive livestock production is commonly associated with high use of veterinary pharmaceuticals, such an area appear to be ideal to get a snapshot of both the internal parasitism and the associated control practices in the country.

Thus, the objective of this study was three-fold: to determine the prevalence and intensity of internal parasite of Bamboutos, to describe the disease control practices in the area, and to evaluate the effect of worm control practices on pig parasitism in the area.

## 2. Materials and Methods

### 2.1. Study Area

**Figure 1:**
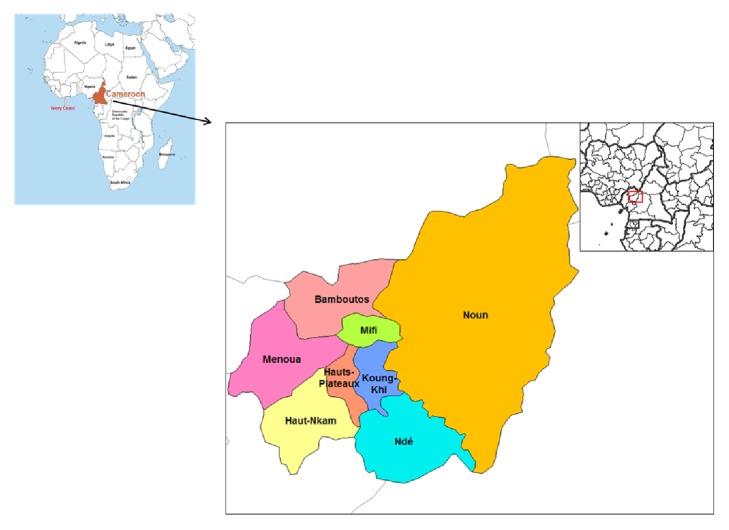
Map showing Bamboutos Division in the West Region of Cameroon.

The study was carried out from May to September 2016 in Bamboutos Division in West region of Cameroon (Figure 1). The area lies between longitude 10°0′-10°30′ east of the Greenwish meridian, and latitude 5°25′-5°50′ north of the equator. The region is characterized by a typical climate with two main seasons, the dry season ranging from November to mid-March, and the rainy season which prevails from mid-March to October. Annual mean temperature is about 20°C and can be as low as 10° in the hills, and the rain fall ranges between 1700 and 2000 mm [17]. Animal husbandry in the division consists of rearing small and large ruminants, cavies, pigs, rabbits, broilers, and layers among others. The west region is one of the highest pig production regions of the country [2].

### 2.2. Study Design and Sample Size Determination

The study was a cross sectional investigation. Small scale pig farms, whether medium sized (population size>8) or small sized (population size ≤8), were selected using the snowball sampling technique. The snowball sampling technique was used due to absence of farmer's registers in the veterinary health authorities' office of the west region. All pigs were sampled in the piggery if the population size was less than 8 animals. Above eight animals on a farm, a maximum of ten animals were randomly sampled. Pregnant sows and piglets below 2 months old were excluded from the study, as well as farms which had received an anthelmintic treatment within 2 months before the study.

The sample size was computed based on the formula for sample size calculation [18] as follows: n = Z^2^P(1 – P)/d^2^, where n is the required sample size, Z is the normal deviate (1·96) at the 5% level of significance, P is the estimated prevalence of parasite infestation in pigs (37.2%) as documented in a previous study in the west region [1], and d is the allowable error of estimation or precision (0·05).Thus, the computed sample size (n) was determined as 359. However, the total number of sampled animals was reduced to 324 due to logistic problems.

### 2.3. Stool Samples and Data Collection

Farms were visited early in the morning before animal defecation. Since the psychosis of African swine fever was present at the time of sampling, the investigators did not come into contact with pigs. Farm owner gently hit the pigs' back to make them defecate. Immediately after defecation, the topper layer of the stool that has not touched the ground was collected with gloved hands and introduced into a screw cap container containing 10% formalin. Data on herd characteristics, herd management practices, farmer status, and worm control practices were collected through a questionnaire. The investigators completed the questionnaire by interviewing the farmers at the time of stool collection.

### 2.4. Fecal Sample Analysis

Faecal samples were analyzed qualitatively and quantitatively using the saturated salt solution (NaCl) as flotation fluid. The simple flotation method was used to detect the parasite eggs and oocysts which were identified microscopically based on morphology and size [4, 11, 19]. The Modified Mc Master [11] test, with a sensitivity of 50 eggs/cyst/oocysts per gram of feces (epg/cpg/opg), was used to estimate the parasitic burden in the individual pig fecal sample.

Heavy eggs were screened using the simple sedimentation test, as described by Zajac and Conroy [11]. Slides were mounted and examined at 100 and 400 magnifications.

An animal was considered infested with a parasite if at least one egg was detected in the flotation solution.

### 2.5. Statistical Analysis

The prevalence was presented in terms of percentage whereas the epg/cpg/opg was presented in terms of mean and standard deviation. The prevalence was computed with the 95% confidence interval (CI). The Chi- squared (*χ*^2^) and Fisher's exact tests were used to determine the association between prevalence of parasites and disease control practices. The association between egg load and disease control practice was investigated using the Mann-Whitney U and the Kruskal–Wallis ANOVA tests. Bonferroni's correction was used in case of multiple comparisons. A p value of <0·05 was considered significant. All statistics were performed using the SPSS statistical package (version 13.0, SPSS Inc., USA)

## 3. Results

The prevalence of internal parasites and the intensity of infestation based on egg/oocyst count are presented in Table 1. Strongylid parasites and four other nematodes, one acanthocephalan and one protozoan, were detected. The overall prevalence was 74.7% (242 out of 324) (95 % CI: 69.6–79.3%) while the overall prevalence of helminthes was 68.2% (221 out 324)( 95% CI: 62-8-73.2%). Strongylid parasites were found in all herds and recorded the highest prevalence (58.6%). The other parasites with high prevalence included coccidia (26.9%) and* Strongyloides ransomi *(25.9%).* Macracanthorhynchus hirudinaceus* recorded the lowest prevalence (0.62%). Other parasites found with low prevalence comprised* Ascaris suum* (3.7%),* Metastrongylus *sp (0.9%), and* Trichuris suis* (0.9%). The overall mean epg for helminths was 129.2±146.5 (range: 50-1400), and the overall mean epg/opg for both helminths and protozoa was 304.1±1218.0 (range: 50-12550). Among helminths, Strongylid parasites shed eggs the most (mean epg = 105.0±134.7, range: 50-1300), followed by* Strongyloides ransomi* (mean epg = 61.9± 40.8). The mean epg was similar for the remaining helminths found.

**Figure 2:**
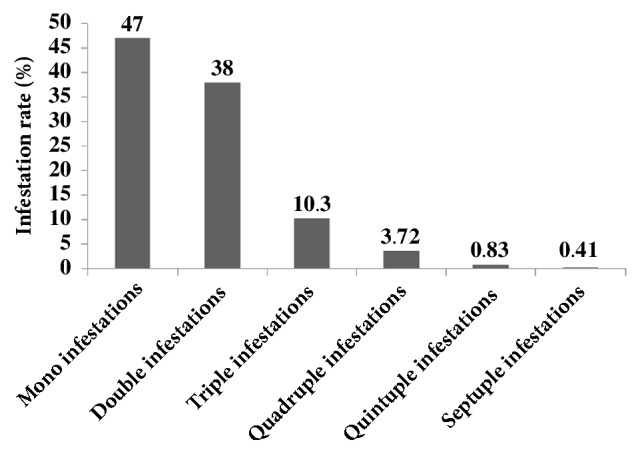
Occurrence of polyparasitism in indoor pigs in Bamboutos, Cameroon.

Pigs with single (47%) and double (38%) infestations were more common, while septuple infestations (0.41%) were the least observed (Figure 2). Strongylid parasites were found in almost all combinations (Table 2).

The data on disease control practices are presented in Table 3. A total of 49 out of 50 (98%) farms sampled had a prophylaxis program. This program consists of general hygiene and use of pharmaceutics. Vaccination was performed in 98% of farms, and the person in charge of pig health was a veterinarian in the majority of farms (64%), then the farmer himself in fewer farms (26%), and both a veterinarian and the farmer in the smallest number of farms (10%). Conventional drugs alone were used in 88% of farms whereas traditional drugs alone were not utilized in any farm. The two drug types were used in 12% of farms. In 66 % of farms, the treatment frequency (deworming) was equal or less than 3 times a year, and the mean annual treatment frequency was 3.82±5.17.

**Figure 3:**
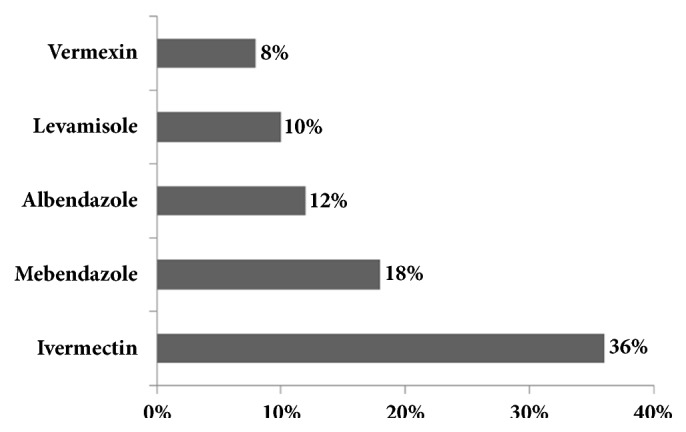
Distribution of indoor pig herds according to anthelmintic usage in Bamboutos, Cameroon.

The distribution of farms per drug used and parasite occurring on the farm is shown in Table 4. Strongylid parasites occurred in all farms, irrespective of the drug used on the farm.* Trichuris suis* and* M. hirudinaceus *occurred only on farms using no other drug than Ivermectin while* A. suum* did not appear in herds treated with Levamisole and Vermexin. The frequency of drug usage is shown in Figure 3. Ivermectin was used in 36% of farms and appeared as the top drug in pig farming in the area, followed by Mebendazole (18%), Albendazole (12%), Levamisole (10%), and Vermexin (8%).

The effect of disease control practices on parasitism is presented in Table 5. The prevalence of Coccidia was associated with the person responsible for animal health while that of S*. ransomi* was associated with the presence of a prophylaxis program and the annual deworming frequency. The prevalence of Coccidia was significantly (p<0.05) higher in animals whose health care depends on a veterinarian than in animals cared for by a farmer. Similarly the prevalence was greater in animals cared for by both the veterinarian and the farmer than in animals cared for by the farmer alone. The prevalence was significantly lower for* S. ransomi* in animals belonging to farms not implementing a prophylaxis program compared with animals reared in farms with a prophylaxis program. Still for this parasite, the prevalence was significantly (p<0.05) higher in pigs dewormed three or less than three times a year compared with animals treated more than three times a year. Overall, animals kept in farms without a prophylaxis program had a significantly (p<0.05) high egg/oocyst load than animals kept in farms with a prophylaxis program. Likewise, pigs dewormed three or less than three times a year significantly (p<0.05) shed more Coccidia oocysts than pigs treated more than three time a year.

## 4. Discussion

Up to three phyla were found to occur in pigs reared under medium sized and small sized farms in Bamboutos. This is in contrast with other reports in sub-Saharan Africa where parasites infesting pigs are limited to nematodes only [20, 21], or both nematodes and protozoa [1, 9]. In studies reporting three phyla [22, 23], nematodes, cestodes, and protozoa often occur, but not acanthocephalan. Only two cases of* M*.* hirudinaceus *were detected in a single farm in this study, and the low prevalence agrees with the finding of a recent study in Uganda [24] showing a prevalence of 2%. Unlike nematodes and protozoa which are characterized by a direct life cycle, acanthocephalan used an indirect life cycle [25]. The difference in the life cycles might explain why acanthocephalan is less common. Strongyles were the predominant parasites occurring in pigs.

The parasite spectrum was similar to that of a previous study in the west region [1], except for the presence of* M*.* hirudinaceus *not previously found. On the contrary, the parasite spectrum was lower than that reported in Plateau State Nigeria [26]. The difference is probably related to the different diagnostic techniques used in both studies. The flotation and sedimentation techniques used in this study, though more sensitive [11], cannot detect protozoan trophozoites while the direct smear used in the study by Agumah et al. [26] can detect protozoan trophozoites and parasite eggs, cysts, and oocysts.

About 75% of pigs were infested with one or more parasite species. This is slightly lower than that documented by Tchoumboue et al. [1] in Cameroon who found an overall prevalence of 97.6%. This indicates that the current use of pharmaceuticals has not greatly impacted pig parasitism in the study site, and possibly in the country. The overall prevalence is similar to that obtained in past studies [22, 23] in Africa but is also higher than that recorded in other studies [26] still in Africa. Differences in diagnostic techniques, eco climatic conditions of the farming area, and management systems could explain the discrepancy in the results. The prevalence of Strongylid parasites was the highest in this study and agrees with other studies [21] and could be due to the damp in piggeries, coupled with the warm temperature in the tropics that provide optimum conditions for strongyle ova development. This finding is contradictory to previous reports [23, 26, 27] in which the prevalence of* A. suum* was the highest; pigs examined in these studies were scavenging pigs while those sampled in this study included indoor pigs. The high resistance of* A. suum *egg in the environment for years [4] explains why* A. suum* is more present in outdoor pigs. The mean egg of Strongylid parasites, though low (<500epg) to moderate (<2000epg), was the highest among helminths probably related to the great number of strongyles (*Hyostrongylus* sp,* Oesophagostomum *sp*, Trichostrongylus *sp, and* Globocephalus* sp) likely to occur in pigs. Coccidia were the only protozoa found, with a prevalence slightly higher than that found by Nonga and Paulo [28] and Karaye et al. [23] who reported a prevalence of 19% and 14%, respectively. Moist and warm environment is conducive for coccidian transmission; this probably justifies why Coccidia and Strongylid parasites were the most prevalent in pigs in this study. The prevalence of* S*. ransomi (25.9%) in this work disagreed with the findings by Esrony et al. [29] in Tanzania and Marufu et al. [20] in Zimbabwe who described a prevalence of 9% and 14 %, respectively. The difference is likely due to the fact that the Tanzanian study site included semiarid areas while in Zimbabwe the temperature in the study site is hotter than in Bamboutos. Such conditions provide unfavorable environment for survival of* Strongyloides* larvae, since these larvae are susceptible to desiccation [4].

Mixed infestations were very common and would surely be causing serious harm in pigs' health and welfare due to the concomitant action of each of these internal parasites of veterinary importance [30].

In the present study, 98% of farmers implemented a prophylaxis program mostly consisting of general hygiene (cleaning, removal of fecal material, and feed refuse each morning), renewal of drinking water, vaccination, deworming, full sanitation of place before the new pig lot, and so forth. The low prevalence of* A. suum *and other helminthes would be the result of this program. However, the high prevalence of Strongylid parasites, and the presence Coccidia and* S. ransomi* suggest that the prophylaxis program is not quite effective against all types of parasites. Since* S. ransomi *may be transmitted through milk during breast feeding, an effective prophylactic measure against* S. ransomi* could be the separation of newborn pigs from infected mothers. This care should be implemented/oriented in future prophylactic programs. The persistent wet environment occurring in piggeries in the study area is known to maintain gastrointestinal transmission [4]. The vaccination was performed in 98% of farms. Though vaccine was mostly against erysipelas, it is believed that vaccination is beneficial against diseases in general due to the strong immunity built by the vaccine (eg: synergism is prevented) and also because of the cross protection incurred by the cross reaction of vaccine. The fact that in 64% of farms a veterinarian was the only animal health care taker and the fact that in 88% of farms conventional drugs alone were used contradict other studies in which farmers are the main animal healers and ethnoveterinary medicine is preferred over conventional medicine [31–33].

This study reports the use of four anthelmintics by farmers. These drugs are all indicated for the treatment of helminths found in this work. Record of helminths on farms is likely the result of re-infestations since only animals that had not received a dewormer within two months were eligible for the study. Moreover, the low mean egg counts observed for helminthes is probably due to anthelmintic treatment*. T. suis* and* M*.* hirudinaceus *occurred only on farms using Ivermectin. Ivermectin may yield variable results against* T. suis* if given in feed [25]; the few farmers with* T. suis* on their farm might have administered Ivermectin in feed. Indeed, 26 % of farmers reported to treat animals themselves. Ivermectin is not indicated against* M. hirudinatus* [25] but dirt and presence of beetle would have caused the single farm to be infested by this parasite. The fact that* A. suum* did not appear in herds treated with Levamisole and Vermexin probably resulted from the low number of farmers using these drugs (10%) compared with the high number using Ivermectin (36%). Ivermectin was the most widely used drug probably because of its systemic effect, being efficient against helminthes and ectoparasite infestations.

Some management practices were found to influence parasitism in this study. The effect of person in charge of animal health on the prevalence of Coccidia was observed, with higher prevalence recorded in farms where a veterinarian is involved in animal health care. This could be attributed to the lax behavior of farmers who neglect the general hygiene of the farm once a veterinarian is involved in their animal health care. The mean Coccidia oocyst count was significantly higher in animals treated more than three times a year. In fact, anthelmintics have no effect on coccidian. Also, the overall mean epg/opg was lower in farms with a prophylaxis program than in farm without a prophylaxis program thus confirming the protective and preventive role of prophylaxis on farm. Treating more than three times a year did not affect the overall mean epg/opg, suggesting that hygiene was more efficient than overuse of anthelmintic in the study area. This confirms the saying that “prevention is better than cure.” Thus overuse of drugs in the study area seemed to be useless, not only because it did not result in positive result regarding parasite load, but especially because high treatment frequency is one of the risk factors for the selection of anthelmintic resistance [34]. Though anthelmintic resistance is seldom reported in pigs, it is worth mentioning that anthelmintic resistance has been documented to be common among* Oesophagostomum* spp. in housed pigs [35].

In conclusion, this study showed that common nematodes, coccidian and acanthocephalan, all of veterinary importance, occur at various prevalence and intensity in pigs in Bamboutos, despite the use of anthelmintics and the implementation of a prophylaxis program by farmers. The majority of farmers resorted to modern veterinary services and mostly used conventional drugs. Knowledge and use of ethnoveterinary medicine was poor in the area. The mean egg/oocyst was low to moderate confirming the use of anthelmintics on pig herds. The occurrence of these internal parasites was associated with some management characteristics. The implementation of a prophylaxis program significantly reduced the overall egg/oocyst load while high treatment frequency did not, indicating that prophylaxis measures such as general hygiene must be reinforced in pig herds in the country, and the treatment frequency reduced as much as possible to prevent selection of anthelmintic resistance.

## Figures and Tables

**Table 1:** Prevalence and intensity of internal parasites of pigs in Bamboutos, Cameroon.

**Parasite**		**Prevalence**			**Intensity**	
	**N**	**n**	%	**95**%**CI**	**Mean epg**±**Sd**	**Range**
**Strongylid parasites**	324	190	58.6	53.1-64.1	105.0±134.7	50-1300
**Coccidia**	324	87	26.9	22.2-32.1	517.2± 1862.1	50-12500
***Strongyloides ransomi***	324	84	25.9	21.3-31.1	61.9± 40.8	50-350
***Ascaris suum***	324	12	3.7	2.0-6.6	50.0±0.0	50
***Metastrongylus sp†***	324	3	0.9	0.2-2.9	50.0±0.0	50
***Trichuris suis***	324	3	0.9	0.2-2.9	50.0±0.0	50
***Macracanthorhynchus hirudinaceus***	324	2	0.62	0.1-2.5	50.0±0.0	50
**All helminths∗**	324	221	68.2	62-8-73.2	129.2±146.5	50-1400
**Overall infestation∗**	324	242	74.7	69.6-79.3	304.1±1218.0	50-12750

N= number of samples examined; n= number of positive samples; CI= confidence interval; epg/opg= egg/oocyst per gram of feces; Sd = standard deviation. *∗*=animals were infested by at least one parasite species; †=*Metastrongylus* sp egg is smaller and contains a larva while strongyle type eggs contain numerous cells.

**Table 2:** Distribution of concomitant infestations in indoor pigs in Bamboutos, Cameroon.

**Number of Parasites**	**Parasites**	**Frequency**	**Percentage**
**n=2**			
	Str1+Str2	16	4.93
	Str1+Str3	2	0.61
	Str1+Strongyloides *ransomi*	30	9.25
	Str1+*Ascaris suum*	1	0.30
	Str1+ *Macracanthorhynchus hirudinaceus*	1	0.30
	Str1+*Trichuris suis*	1	0.30
	Str1+Coccidia	32	9.87
	*Strongyloides ransomi* +*Ascaris suum*	1	0.30
	*Strongyloides ransomi* + *Macracanthorhynchus hirudinaceus*	1	0.30
	*Strongyloides ransomi* +Str2	4	1.23
	*Strongyloides ransomi* +Coccidia	1	0.30
	*Ascaris suum*+Coccidia	2	0.61
**n=3**			
	Str1+Str2+Coccidia	4	1.23
	Str1+*Stongyloides ransomi* +Coccidia	17	5.24
	Str1+Str2+*Strongyloides ransomi*	1	0.30
	Str1+*Strongyloides ransomi* +Metastrongylus	1	0.30
	Str1+*Metastrongylus* sp+Coccidia	1	0.30
	Str1+*Trichuris suis*+ Coccidia	1	0.30
**n=4**			
	Str1+Str2+*Strongyloides ransomi* +Coccidia	2	0.61
	Str1+*Strongyloides ransomi* +*Ascaris suum*+Coccidia	1	0.30
	Str1+*Strongyloides ransomi* +*Metastrongylus* sp+Coccidia	1	0.30
	Str1+Str2+*Strongyloides ransomi* +*Ascaris suum*	1	0.30
	Str1+*Strongyloides ransomi* +*Trichirus suis*+Coccidia	1	0.30
**n=5**			
	Str1+Str2+Str3+*Strogyloides ransomi* +*Ascaris suum*	2	0.61
**n=6**			
	Str1+Str2+Str3+*Strongyloides ransomi*+*Ascaris suum*+Coccidia	1	0.30

Str1: strongyle type 1; Str2: strongyle type 2; Str3: strongyle type 3.

**Table 3:** Distribution of pig herds according to disease control practices in Bamboutos, Cameroon.

**Practices**	**Number of studied herds**	**Herds undergoing disease ** **control practices**	
		n	%
**Prophylaxis program exists**	50	49	98
**Vaccination is done**	49	48	98.0
**Animal health caretaker is**			
a veterinarian	50	32	64.0
the farmer himself	50	13	26.0
both	50	5	10.0
**Drug type**			
Conventional alone	50	44	88.0
Ethno-veterinary alone	50	0	0
Both	50	6	12.0
**Treatment frequency per year**			
≤3	50	33	66.0
> 3	50	17	34.0
*mean±sd *	*50*	*3.82±5.17*	

**Table 4:** Distribution of pig herds according to type of drug used and parasites present on farm in Bamboutos, Cameroon.

**Parasite**	**Drug**								
	Iver	Dew*∗*	Meb	Lev	Ver†	Alb	M+L	A+L	A+L+M
	N=18	N=10	N=8	N=2	N=4	N=4	N=1	N=1	N=1
**Strongylid parasites**	18(100.0)	10 (100.0)	8(100.0)	2(100.0)	4(100.0)	4(100.0)	1(100.0)	1(100.0)	1(100.0)
**Coccidia**	3(16.7)	2(20.0)	2(25.0)	1(50.0)	0(0.0)	0(0.0)	1(100.0)	0(0.0)	0(0.0)
***Strongyloides ransomi***	13(72.2)	8(80.0)	4(50.0)	0(0.0)	2(50.0)	1(25.0)	1(100.0)	0(0.0)	1 (100.0)
***Ascaris suum***	3(16.7)	3 (30.0)	1(12.5)	0(0.0)	0(0.0)	1(25.0)	0(0.0)	0(0.0)	0(0.0)
***Metastrongylus sp***	0(0.0)	0(0.0)	1(12.5)	1(50.0)	0(0.0)	0(0.0)	1(100.0)	0(0.0)	0(0.0)
***Trichuris suis***	2 (11.1)	0(0.0)	0(0.00	0(0.0)	0(0.0)	0(0.0)	0(0.0)	0(0.0)	0(0.0)
***Macracanthorhynchus hirudinaceus***	1(5.6)	0(0.0)	0(0.0)	0(0.0)	0(0.0)	0(0.0)	0(0.0)	0(0.0)	0(0.0)

N= number of herds using a particular drug; Iver= Ivermectin; Meb= Mebendazole; Lev= Levamisole; Ver= Vermexin; Alb= Albendazole; M+L= Mebenzole+Levamizole; A+L= Albendazole+ Levamisole; A+L+M= Albendazole+Levamisole+Mebendazole. Dew= dewormer. *∗*: farmers used dewormers but could not recall their identities. †: Vermexin belongs to the Benzimidazole class of anthelmintic drugs.

**Table 5:** Effect of disease control practices on parasite prevalence (%) and load (mean epg/opg) in pigs in Bamboutos, Cameroon.

**Control practices**	**Strongylid parasites**		**Coccidia**		***Strongyloides ransomi***		**Overall infestation** **∗**	
	n (%)	Mean ± Sd	n (%)	Mean ± Sd	n (%)	Mean ± Sd	n (%)	Mean ± Sd
**Prophylaxis program exists**								
Yes	185 (58.5)	103.8±135.1	84 (26.6)	96.4±174.4	78 (24.7)^a^	62.8±42.2	235 (74.4)	225.3^a^± 1072.2
No	5 (62.5)	150.0±122.5	3 (37.5)	66.7±76.4	6 (75.0)^a^	50.0±0.0	7 (87.5)	325.0^a^± 358.6
**Vaccination**								
Yes	182 (58.1)	104.7±136.0	82 (26.2)	536.6±1915.5	78 (24.9)	-	232(74.1)	226.6± 1077.3
No	3 (100)	50.0±0.0	2 (66.7)	50.0±0.0	0 (0.0)	-	3 (100)	100.0± 50.0
**Health caretaker**								
Veterinarian	128 (61.5)	100.0±94.5	63 (30.3)^a^	547.6±1986.3	57 (27.4)	63.2±44.8	158 (76.0)	256.9± 1167.3
Farmer	48 (59.3)	111.5±213.2	12(14.8)^a,b^	675.0±2149.4	19 (23.5)	63.2±36.7	56 (69.1)	190.1± 986.9
Both	14 (40.0)	128.6±105.1	12(34.3)^b^	200.0±330.9	8 (22.9)	50.0±0.0	28 (80.0)	141.4± 275.8
**Annual treatment frequency**								
≤than 3	117 (56.0)	91.9±96.0	57 (27.3)	73.7±103.6^a^	61 (29.2)^a^	59.0±28.1	157 (75.1)	100.9± 130.9
>3	73 (63.25)	126.0±178.9	30 (26.1)	1360.0±3023.3^a^	23 (20.0)^a^	69.6±63.5	85 (73.9)	458.3± 1752.4

epg/opg= egg/oocyst per gram of feces; Sd = standard deviation.

^abc^: values with similar superscripts for a given practice within a column are significantly different (p<0.05).

*∗*: for overall infestation, the “n” represents the number of animals infested by at least one parasite species.

## Data Availability

The data used to support the findings of this study are included within the article.
